# Book Notices

**Published:** 1891-10

**Authors:** 


### Book Notices.

TEXT-BOOK OF OPHTHALMOSCOPY. By Edward G. Loring, M. D. Edited by Francis B. Loring, M. D. Part II. Diseases of the retina, optic nerve, and choroid, their varieties and complications. 253 pages. New York: D. Appleton & Co., 1, 3 and 5 Bond street, 1881.

This is the last work of Dr. E. G. Loring, who died suddenly in April, 1888, before finishing the book. Very little if any changes have been made in the text by the editor, Dr. F. B. Loring. It is a work of merit, and there is much that is original in it. The book contains seven chapters. The I. discusses the vascular disturbances of the retina; II. Irritation of the retina; III. Inflammation of the retina; IV. Diffuse retinitis, microscopic characteristics, etc., etc.; V. Diseases of the optic nerve, etc.; VI. Atrophy of the optic nerve, etc.; VII. Affections of the chloroid, etc. B.

---

MODERN METHODS OF ANTISEPTIC WOUND TREATMENT, is the title of a most interesting and handsomely illustrated pamphlet issued recently by Johnson & Johnson, the well known manufacturing druggists, of New York. It is an exhaustive—though not an exhausting—review of the progress which has been made in the treatment of Wounds on the antiseptic principle, and especially through the agency of fixed antiseptic dressings. In this work are pointed out all the earlier defects of technique, and the methods now found most satisfactory are fully explained. These manufacturers keep fully abreast of the most advanced ideas on dressings, and put them in shape, ready to use at a moments notice. The pamphlet will be mailed free on application, if DANIEL'S TEXAS MEDICAL JOURNAL be mentioned.

---

MATERIA MEDICA AND THERAPEUTICS, with special reference to the clinical application of drugs. By John V. Shoemaker, A. M., M. D.; Professor of Materia Medica, Pharmacology, Therapeutics, and Clinical Medicine, Etc., in the Medico Chirurgical College, Philadelphia.

Being the second and last volume of a treatise on Materia Medica, Pharmacology and Therapeutics. An independent volume upon drugs. The first volume of this work came out last year and was favorably noticed in this journal. The second, now before us, was anxiously waited for. One of the chief features of this volume is its reference to new remedies. A great many works on this subject ignore all new agents because they have not been sufficiently tested, but at this day and time when books are made so readily, one feels disappointed in not finding the latest remedies discussed up to date. The older and more thoroughly tested remedies, of course, have the greater emphasis.

The work is well arranged and eminently scientific. B.

